# Prevalence and risk of skeletal complications and use of radiation therapy in elderly women diagnosed with metastatic breast cancer

**DOI:** 10.1371/journal.pone.0193661

**Published:** 2018-03-01

**Authors:** Arif Hussain, Candice Yong, Katherine H. R. Tkaczuk, Yi Qian, Jorge Arellano, C. Daniel Mullins, Eberechukwu Onukwugha

**Affiliations:** 1 Department of Medicine, University of Maryland Medical Center, Baltimore, MD, United States of America; 2 Veterans Affairs Medical Center, Baltimore, MD, United States of America; 3 University of Maryland School of Pharmacy, Baltimore, MD, United States of America; 4 Amgen Inc., Thousand Oaks, CA, United States of America; University of South Alabama Mitchell Cancer Institute, UNITED STATES

## Abstract

**Objective:**

Real-world data regarding patient factors associated with the occurrence of spinal cord compression (SCC) or pathological fracture (PF), or need for bone surgery (BS), or use of radiation therapy (RAD) (i.e. skeletal complications and radiation therapy; SCRT) are limited for women with metastatic breast cancer (BCa). Given the substantial clinical and economic burden of these events in advanced BCa, we conducted the present study to understand the prevalence and identify the risk factors associated with these events among elderly women presenting with de novo metastatic BCa.

**Methods:**

Using linked Surveillance, Epidemiology, and End Results and Medicare data, we identified women with incident metastatic BCa diagnosed during 2005–2009. Associations between patient demographics and select clinically relevant factors, and SCRT were examined using the Cox proportional hazards model, accounting for death as a competing risk.

**Results:**

Of 3,731 Medicare beneficiaries with incident metastatic BCa, 1,808 (48.5%) experienced at least one SCRT event during a median follow-up of 13.2 months; a majority (69%) experienced a subsequent SCRT event. The proportions of women who had RAD, PF, BS, and SCC were: 32%, 28%, 8%, and 4%. Older women (80+ years), or those with more comorbid conditions (CCI≥2) had a statistically significant lower risk of SCRT (HR 0.78 [CI: 0.67–0.92, p<0.01]; HR 0.77 [CI: 0.67–0.89, p<0.01], respectively), primarily due to lower frequency of radiotherapy (p<0.01). Compared to Caucasians, African Americans had lower risk of SCRT (HR 0.70 [CI: 0.60–0.82, p<0.01]), as well as all SCRT subtypes defining this group except for SCC, which was the same for both race groups.

**Conclusion:**

This study highlights that certain patient characteristics and clinical factors are associated with the risk of spinal cord compression or pathologic fractures, or need for bone surgery or radiation among women with metastatic BCa. In future studies, it will also be important to consider the clinical and economic burden based on these components of skeletal complications and radiation therapy use in order to guide and improve the management of women with advanced BCa.

## Introduction

Clinically relevant complications due to bone metastasis among patients with malignancies are termed skeletal related events (SREs), and include a composite of the following entities: spinal cord compression (SCC), pathologic fracture (PF), bone surgery (BS), and radiation therapy to the bone (RAD) for pain relief or to prevent fractures. In the United States breast cancer (BCa) is the most common type of cancer diagnosed in women, with the majority presenting with early stage, generally curable disease. De novo presentation of advanced BCa is becoming increasingly uncommon, but among those who do or among those who eventually develop metastatic disease, the presence of SREs is associated with increased mortality [1, 2], considerable healthcare resource utilization and costs [3–6], and reduced health-related quality of life, including impaired mobility and functional status [7]. For instance, based on U.S. SEER (Surveillance, Epidemiology, and End Results)-Medicare data on 98,260 older women with BCa, when compared to women without any bone metastasis the hazard ratio for risk of death was 4.9 (95% CI: 4.7–5.1) among women with bone metastasis but no SRE and 6.2 (95% CI: 5.9–6.5) among women with bone metastasis plus SRE [1].

Evidence regarding the incidence of SREs in BCa is drawn largely from randomized clinical trials. Data from these trials that evaluated the efficacy of several bone-modifying agents, including pamidronate, zoledronic acid, and denosumab, indicate that SREs occurred in 30%-56% of women with BCa and bone metastasis in the intervention arms, and 50%-67% among those in the placebo arms [8–11]. Given the positive impact of these agents in decreasing the incidence of bone-related SREs, the U.S. Food and Drug Administration approved these anti-resorptive drugs for the treatment of patients with bone metastases due to BCa. While there have been several recent observational studies reporting the clinical and economic burden of SREs in other cancers, including that of the prostate, lung, and thyroid, as well as multiple myeloma[12–16], there is a paucity of more recent real-world evidence relating to the prevalence and burden of skeletal complications in metastatic BCa.

The median age at diagnosis of BCa in the U.S. is 62 and 43% of BCa cases are diagnosed in women aged 65 years and older [17]. However, information on the burden of skeletal related complications in older women with advanced BCa is limited [4]. Moreover, while previous studies have examined the prevalence, mortality and economic impact of SREs, there is less data on the prevalence of SRE subtypes or the risk factors associated with SRE occurrence among older women with incident metastatic BCa.

Given the limited information on skeletal metastasis-related complications in BCa in a real world setting, we undertook the present study utilizing the SEER cancer registry linked to Medicare claims data to gain a greater understanding of the factors that potentially increase the risk of SCC, PF, BS and the use of radiation among a broader population of women presenting with advanced BCa than typically defined within the context of clinical trials. Starting in 2004, the Collaborative Staging System has been applied to cancer cases in SEER. In addition to information on tumor size, extension and lymph node status if available, the Collaborative Staging System provides information on the site of metastasis at diagnosis. In contrast to prostate cancer where, starting in 2004, patients with incident metastatic (M1) disease included within the SEER database are further categorized into M1a (distant lymph node metastasis), M1b (bone metastasis) or M1c (metastasis to other organs) subgroups, the Collaborative Staging System for BCa does not have a data field that identifies metastasis to the bone alone. Given this, any radiotherapy used in women with advanced BCa within the SEER database could be related to their having bone metastasis per se, or possibly for some other indications which may or may not be related to bone metastases. Thus, there is a potential for some misclassification in terms of radiation, given that we define and understand the use of radiation in the context of SREs as being representative of an intervention related to managing skeletal metastasis. Nevertheless, understanding the use of radiation in an aggregate metastatic BCa patient population as defined within SEER would still be informative in further clarifying the patterns of use of radiation with respect to patient and clinical characteristics in a more ‘real world’ setting.

Given the above considerations, the objectives of our study were to determine the prevalence of events (SCC, PF) and interventions (BS and radiation use) in elderly women presenting with de novo metastatic (M1) BCa. In the present study, the clinical elements of SCC, PF, BS and radiation use are referred to collectively as skeletal complications and radiation therapy (SCRT) hereafter since this potentially represents a broader category than is typically defined as SREs in clinical trials.

## Materials and methods

### Study design and study sample

This study was conducted using linked Surveillance, Epidemiology, and End Results cancer registry and Medicare claims data (SEER-Medicare). The study included de-identified data, and was approved by the University of Maryland Baltimore Institutional Review Board (IRB) with a waiver of consent. It included women aged 66 and older diagnosed with incident stage IV breast cancer (BCa) between 2005 and 2009, with Medicare claims data from 2004 to 2010. Stage of BCa was determined based on the American Joint Committee on Cancer Tumor-Node-Metastasis (AJCC-TNM) staging, 6^th^ edition.[18] We limited the sample to beneficiaries with continuous enrollment in Medicare Parts A and B for the 12 months immediately prior to and including the month of diagnosis to be included in the study sample. Patients were excluded if they had the following: 1) history of cancer within 5 years prior to the BCa diagnosis; 2) unknown diagnosis month or year; or 3) incident post-mortem BCa diagnosis.

### Measures

The outcome of interest was SCRT that occurred concurrently with or following diagnosis of metastatic BCa, measured by the presence of claims indicating SCC, PF, BS, or RAD during follow-up. A 21-day window was used to identify unique events for the same type of SCRT, i.e., if the same event occurred more than once within 21 days of each other then it was counted as a single event. We examined the prevalence of any SCRT and each SCRT subtype, as well as the time from diagnosis of metastatic BCa to the first SCRT occurrence. If multiple events occurred on the same day, a hierarchy of SCC, followed by PF, BS, and RAD, was applied to determine the first SCRT type.

Patient-level demographic characteristics, as well as baseline clinical characteristics, including ER (estrogen receptor) and PR (progesterone receptor) status, but not HER2 status (which was not available), were obtained from the SEER PEDSF (Patient Entitlement and Diagnosis Summary) file. We also assessed comorbid conditions at baseline using the Charlson Comorbidity Index (CCI), which was calculated using claims from the 12-month period prior to the month of diagnosis. Starting in 2004, the Collaborative Staging System was applied to cancer cases in SEER. In addition to information on tumor size, extension, and lymph node status if available, the Collaborative Staging System provided information on the site of metastasis at diagnosis. For BCa the Collaborative Staging System does not have a data field that identifies metastasis to the bone alone, but instead includes a composite measure that indicates metastasis to several sites at the time of diagnosis, including bone, adrenal gland, contralateral breast, lung, ovary, or satellite nodule(s) in skin other than primary breast. Using the composite measure available from the Collaborative Staging System, we included a binary indicator for the presence of incident metastasis to the bone (among other sites) in the regression model for estimating the risk of SCRT.

### Statistical analysis

Descriptive characteristics were presented using frequency distributions for categorical variables and mean/median values for continuous variables. Chi-square tests were used to determine the unadjusted association between patient characteristics and any SCRT occurrence. The adjusted association between patient demographic and clinical factors and the hazard of a SCRT was quantified using the Cox proportional hazards model, accounting for death as a competing risk. In the presence of a competing risk, the traditional Cox proportional hazards model would overestimate the hazard of the event of interest because the competing event leads to a censored observation in the analysis.[19, 20] Statistical tests were two-tailed and the cut-off value for statistical significance was 0.05. All statistical analyses were conducted using Version 9.3 of the SAS System.

## Results

**Fig 1:**
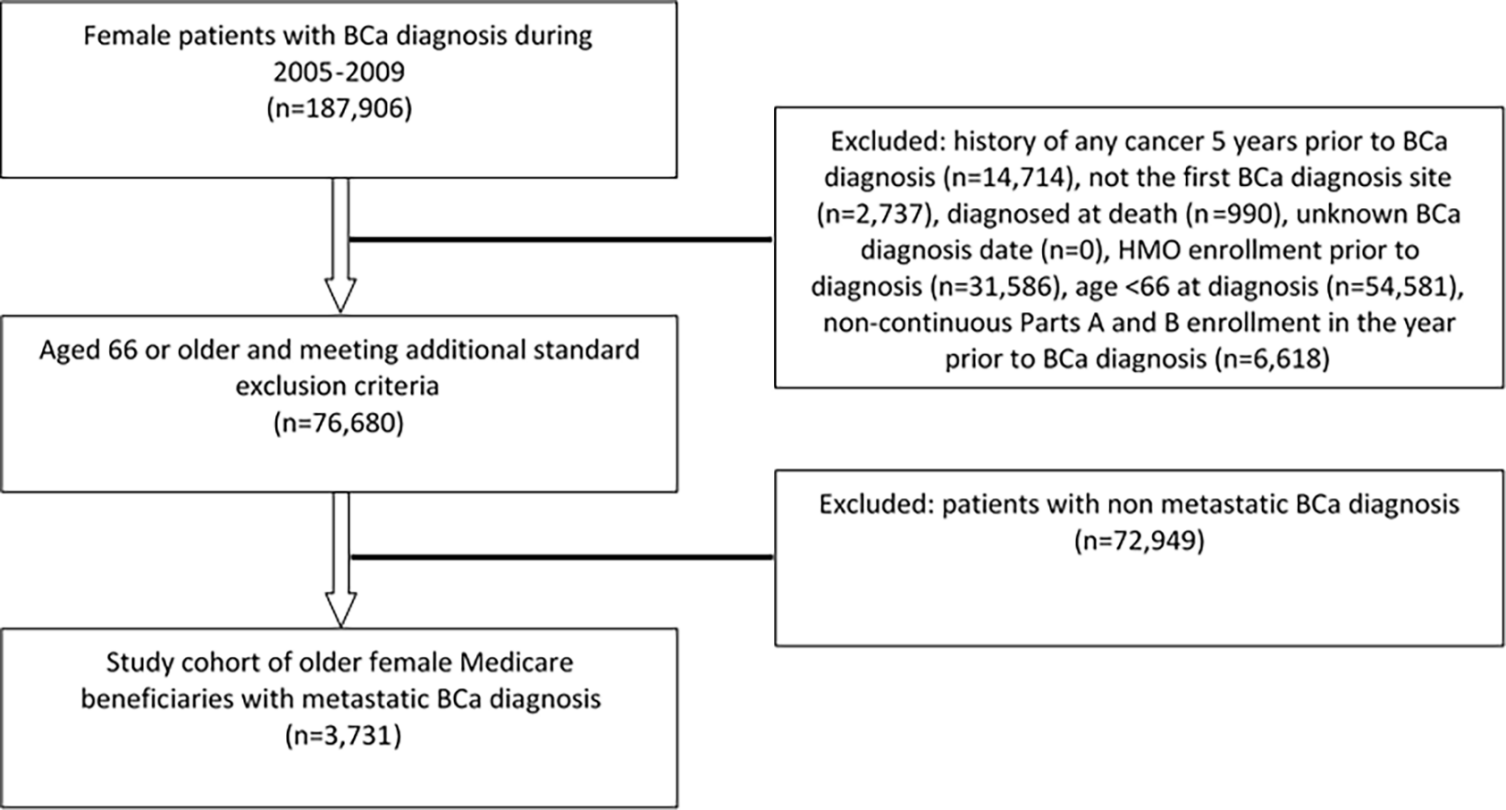
Cohort selection of patients with metastatic BCa diagnosis (N = 3,731).

The study sample included 3,731 older Medicare-eligible women who presented with metastatic BCa between 2005 and 2009 (Fig 1). The median age of the sample was 77 years (IQR: 71–83 years; range: 66–102 years). Mortality at 12 months after diagnosis was 45.7%, while overall mortality was 72% over a median (mean) follow-up period of 13.2 (18.2) months. Table 1 shows the descriptive characteristics of the study sample, stratified by SCRT occurrence.

**Table 1:** Descriptive statistics for women diagnosed with metastatic breast cancer in 2005–2009, by occurrence of spinal cord compression, pathological fracture, bone surgery, or radiation therapy (SCRT) during follow-up (N = 3,731).

	Full Sample		Any SCRT		No SCRT		P-value
	(N = 3,731)		(N = 1,808)		(N = 1,923)		
	N	Col %	N	Col %	N	Col %	
**Age**							<0.01
66–69	661	17.7	362	20.0	299	15.6	
70–74	817	21.9	432	23.9	385	20.0	
75–79	788	21.1	400	22.1	388	20.2	
80–84	734	19.7	312	17.3	422	21.9	
85+	731	19.6	302	16.7	429	22.3	
**Race**							<0.01
Non-Hispanic White	2994	80.3	1486	82.2	1,508	78.4	
Non-Hispanic African American	460	12.3	178	9.9	282	14.7	
Hispanic	161	4.3	89	4.9	72	3.7	
Other	116	3.1	55	3.0	61	3.2	
**ER/PR status**							<0.01
ER/PR positive	1682	45.1	882	48.8	800	41.6	
ER/PR negative	675	18.1	331	18.3	344	17.9	
ER positive/PR negative	494	13.2	266	14.7	228	11.9	
ER negative/PR positive	35	0.9	17	0.9	18	0.9	
ER/PR unknown	845	22.7	312	17.3	533	27.7	
**SEER measure of metastasis to bone and other sites at diagnosis**^**a**^	1504	40.3	799	48.5	705	36.7	<0.01
**Charlson Comorbidity Index**							<0.01
Zero	1956	52.4	998	55.2	958	49.8	
One	721	19.3	360	19.9	361	18.8	
Two or higher	629	16.9	254	14.1	375	19.5	
Missing	425	11.4	196	10.8	229	11.9	

SEER, Surveillance, Epidemiology, and End Results.

^a^A composite measure from SEER that indicates metastasis to several sites at the time of diagnosis, including bone, adrenal gland, contralateral breast, lung, ovary, or satellite nodule(s) in skin other than primary breast.

**Fig 2:**
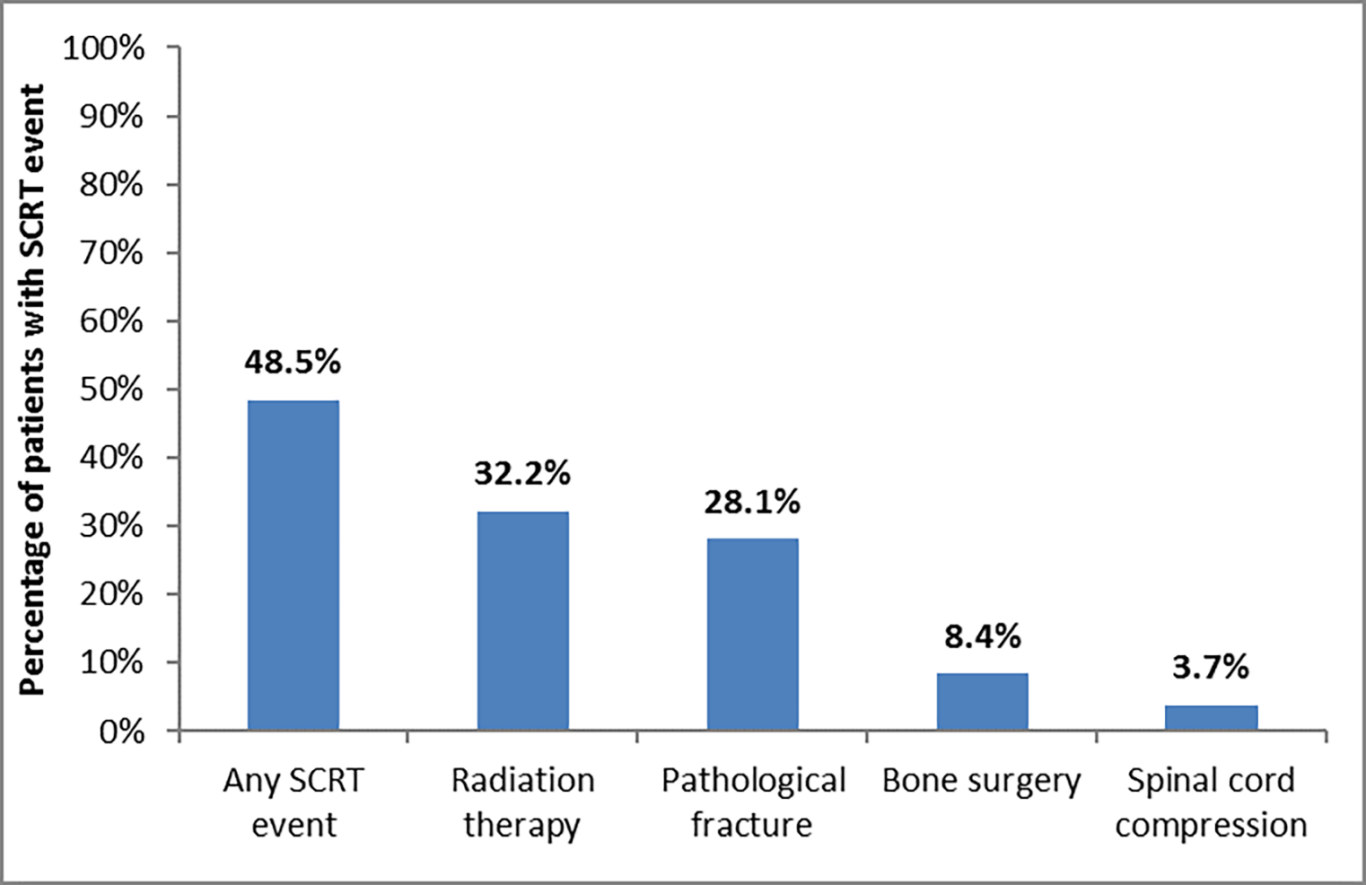
Prevalence of any SCRT event (spinal cord compression, pathological fracture, bone surgery, or radiation therapy) and each SCRT subtype among women with metastatic breast cancer (N = 3,731).

During follow-up, 1,808 (48.5%) of 3,731 patients experienced at least one SCRT. The percentage of women who had any radiation, fracture, bone surgery, or spinal cord compression, were 32.2%, 28.1%, 8.4%, or 3.7%, respectively (Fig 2). Of the 1,808 women who experienced at least one SCRT, the majority had radiation (50%) or fracture (45%) as their first SCRT. A large proportion (69%) of women with one SCRT developed a subsequent SCRT. Among women with at least 1 SCRT, the median (mean) time to first SCRT was 62 (181) days.

Adjusted sub-hazard ratios (HR) for the risk of SCRT occurrence are shown in Table 2. Compared to women aged 66 to 69, the HR (95% CI) for women aged 80 to 84 was 0.78 (0.67–0.92) and for those aged 85 and over it was 0.79 (0.67–0.92). African American women were statistically significantly less likely to experience SCRT compared to women who were non-Hispanic White (HR = 0.70; 95% CI = 0.60–0.82). Compared to women with Charlson comorbidity index (CCI) = 0, women with CCI ≥ 2 were less likely to experience SCRT (HR = 0.77; 95% CI = 0.67–0.89). Also, women in whom the ER/PR status was unknown had statistically significantly lower risk of SCRT compared to those with ER/PR positive metastatic BCa (HR = 0.68; 95% CI = 0.60–0.78).

**Table 2:** Covariate-adjusted sub-hazard ratios (SHR) for occurrence of spinal cord compression, pathological fracture, bone surgery, or radiation therapy (SCRT) among women diagnosed with stage IV breast cancer (N = 3,731).

	SHR	95% CI	p-value
**Age**			
66–69	Reference		
70–74	0.99	(0.86–1.13)	0.89
75–79	0.96	(0.83–1.10)	0.56
80–84	0.78	(0.67–0.92)	<0.01
85+	0.79	(0.67–0.92)	<0.01
**Race**			
Non-Hispanic White	Reference		
Non-Hispanic African American	0.70	(0.60–0.82)	<0.01
Hispanic	1.22	(0.99–1.51)	0.07
Other	0.95	(0.72–1.26)	0.74
**ER/PR status**			
ER/PR positive	Reference		
ER/PR negative	0.92	(0.81–1.04)	0.18
ER positive/PR negative	1.04	(0.90–1.19)	0.63
ER negative/PR positive	0.93	(0.55–1.58)	0.79
ER/PR unknown	0.68	(0.60–0.78)	<0.01
**Charlson Comorbidity Index**			
Zero	Reference		
One	0.97	(0.86–1.09)	0.62
Two or higher	0.77	(0.67–0.89)	<0.01
Missing	0.90	(0.77–1.06)	0.21
No metastasis to bone and other sites at diagnosis^a^	Reference		
SEER measure of metastasis to bone and other sites at diagnosis^a^	1.27	(1.15–1.40)	<0.01

The regression models also controlled for osteoporosis pre-diagnosis, any BMD test pre-diagnosis, region of SEER registry and year of diagnosis.

^a^ A composite measure from SEER that indicates metastasis to several sites at the time of diagnosis, including bone, adrenal gland, contralateral breast, lung, ovary, or satellite nodule(s) in skin other than primary breast.

We further examined the prevalence of SCRT *subtypes* by age group, race, CCI category, and ER/PR status (Table 3). The proportion of women with any radiation was 41.8% among 66–69 year-olds, and decreased steadily to 19.2% among women 85+ year-olds (p<0.01). In contrast, the proportion of women experiencing fracture, bone surgery, or SCC was not statistically different across the various age groups. Hence, the lower risk of SCRT occurrence among older women (aged 80+) compared to younger women (aged 66–69 years) appears to be primarily driven by lower utilization rates of radiation therapy among the older age group. Regarding the different SCRT by race, except for SCC, all other SCRT subtypes were lower among African American women than the other race groups. In terms of CCI, the median follow-up of patients decreased with increasing CCI scores; median follow-up for patients with CCI = 0, 487 days; CCI = 1, 230 days; CCI ≥2, 230 days, respectively. The proportion of patients having radiation therapy, bone surgery or SCC was significantly lower among those with high (CCI ≥ 2) compared to patients with lower CCI scores (Table 3).

**Table 3:** Prevalence of SCRT (spinal cord compression, pathological fracture, bone surgery, or radiation therapy) subtypes by age group, race group, Charlson comorbidity index (CCI) score, and ER/PR status at diagnosis.

**AGE GROUP**						
	**Any SCRT**	**RAD**	**PF**	**BS**	**SCC**	**1-year mortality**
66–69 (n = 661)	54.8%	41.8%	26.5%	7.1%	5.0%	32.2%
70–74 (n = 817)	52.9%	39.3%	28.2%	9.7%	4.5%	40.5%
75–79 (n = 788)	50.8%	35.7%	28.2%	9.0%	3.6%	44.3%
80–84 (n = 734)	42.5%	24.9%	27.1%	6.8%	3.4%	51.0%
85+ (n = 731)	41.3%	19.2%	30.5%	9.0%	2.2%	59.9%
p-value*	< .0001	< .0001	0.51	0.18	0.05	< .0001
**RACE GROUP**						
	**Any SCRT**	**RAD**	**PF**	**BS**	**SCC**	**1-year mortality**
Non-Hispanic White (n = 2,994)	49.6%	32.9%	29.7%	8.9%	3.7%	44.7%
Non-Hispanic African American (n = 460)	38.7%	25.0%	17.4%	4.6%	3.9%	55.0%
Hispanic (n = 161)	55.3%	36.0%	30.4%	9.3%	NR	43.5%
Other (n = 116)	47.4%	36.2%	26.7%	10.3%	NR	37.9%
p-value*	< .0001	0.004	< .0001	0.02	0.97	0.0001
**CCI SCORE**						
	**Any SCRT**	**RAD**	**PF**	**BS**	**SCC**	**1-year mortality**
Zero (n = 1,956)	51.0%	35.9%	28.9%	9.6%	4.1%	40.3%
One (n = 721)	49.9%	31.5%	30.0%	8.3%	3.7%	46.3%
Two or higher (n = 629)	40.4%	21.6%	25.4%	5.4%	1.8%	59.0%
Missing (n = 425)	46.1%	31.8%	25.4%	7.3%	4.9%	49.7%
p-value*	< .0001	< .0001	0.14	0.01	0.03	< .0001
**ER/PR STATUS**						
	**Any SCRT**	**RAD**	**PF**	**BS**	**SCC**	**1-year mortality**
ER/PR positive (n = 1682)	52.4%	36.2%	29.6%	9.2%	4.5%	32.2%
ER/PR negative (n = 675)	49.0%	36.4%	23.1%	6.5%	0	59.4%
ER positive/PR negative (n = 494)	53.9%	37.9%	32.0%	7.7	3.9%	35.2%
ER negative/PR positive (n = 35)	48.6%	NR	NR	NR	NR	71.4%
ER/PR unknown (n = 845)	36.9%	17.6%	26.9%	8.5%	3.2%	66.8%
p-value*	< .0001	< .0001	<0.01	0.18	0.15	< .0001

RAD, radiation therapy; PF, pathological fracture; BS, bone surgery; SCC, spinal cord compression;

NR, Not reported due to small cell size (<11), as per data use agreement.

* P-value based on Chi-square test.

With regards to ER and PR, in almost 23% of the women the ER/PR status was unknown/undocumented in the SEER dataset. The median follow-up time for the ER/PR unknown women was considerably shorter (148 days) than for ER/PR positive (590 days), ER positive/PR negative (527 days) and ER/PR negative (226 days) women. The one-year mortality was also significantly higher for the ER/PR unknown patients (66.8%) compared to those with known ER/PR status (32.2–35.2%) (p<0.01). The ER/PR unknown women were significantly less likely to receive radiation therapy (p<0.01; Table 3).

## Discussion

Over 266,000 women are estimated to be diagnosed with invasive ductal breast carcinoma in the U.S. in 2018.[21] Although the majority of women present with clinically localized disease which is generally treatable, metastatic breast cancer remains incurable, with over 40,900 deaths projected to occur due to advanced breast cancer in 2018.[21] Women with metastatic breast cancer include both those who present de novo with metastatic disease (6–10% incidence) and those who develop recurrent progressive disease after their initial diagnosis. Thus, although the incidence of metastatic breast cancer at presentation is relatively low, this group constitutes a significant proportion of the women dying of breast cancer. Skeletal metastasis represents among some of the most clinically significant complications of metastatic breast cancer that negatively impacts clinical outcomes, including morbidity and mortality, and puts significant burden on health services. Thus, considerable efforts have been made to mitigate the clinical impact of skeletal metastasis, termed skeletal related events, via well-defined prospective clinical trials in women with documented skeletal metastasis.

The present study was carried out in an effort to identify the clinical and pathological risk factors that may be associated with and potentially relevant to the occurrence of spinal cord compression or pathologic fracture, or need for bone surgery, or use of radiation (collectively termed SCRT) in a broader population of newly diagnosed advanced BCa patients seen in a ‘real-world’ setting. Using the SEER registry as a model of such a ‘real world’ patient population, we conducted the present study in women with incident diagnosis of metastatic BCa between 2005 and 2009, with a particular emphasis on demographic and clinical factors that may be associated not only with the composite category termed SCRT but also the specific subtypes that make up this broader category.

Approximately one in two women with incident advanced BCa experienced at least one SCRT at a median follow up time of 13.2 months. Among those with any SCRT, the majority (69%) experienced a subsequent SCRT. The most common SCRT events were radiation therapy and bone fractures. Somewhat unexpectedly, several patient-related factors emerged as being associated with a statistically significant lower risk of having a SCRT, and included older age, AA race, CCI≥2, and unknown status of ER/PR expression.

Our analysis of SCRT subtypes demonstrated that less use of radiation was the primary driver for the overall lower prevalence of SCRT among the older age groups. We noted a significant increase in the risk of 1 year mortality in women with higher CCI score, (40.3% for CCI = 0 vs. 59% for CCI≥2). Again, it appears that a main driver for the lower prevalence of SCRT among women with CCI scores ≥ 2 was significantly less radiation therapy, while bone surgery and SCC also contributed, but to a lesser extent, to the composite SCRT category (Table 3). The current definition of SCRT includes both treatments (radiation and/or surgery) and events (fracture and spinal cord compression). Taken together, our results suggest that women with metastatic BCa who are older and/or in poorer health are particularly less likely to receive treatments such as radiation therapy often used to palliate and control complications associated with advanced BCa in younger and healthier patients.

Although the median age of AA and Non-Hispanic White (NHW) women were similar (75 vs 77 years, respectively), the former experienced a greater comorbidity burden, had potentially more aggressive disease (as reflected by the proportion of women with ER/PR negative cancer), had higher mortality and a shorter median follow-up. More specifically, 37.6% of AA vs 55.4% of NHW women had CCI = 0, while 23.3% of AA vs 15.6% of NHW women had CCI ≥ 2 (p<0.01). In terms of ER/PR status, 26.7% of AA vs 16.6% of NHW women were ER/PR negative BCa (p<0.01), while one-year mortality was 55% for AA women vs 44.7% for NHW women (p<0.01). Despite such differences, AA women had significantly less fractures (p<0.01); such racial differences in fracture risk are also observed among men with metastatic prostate cancer.[22] Thus, AA patients with advanced hormonally driven cancers (such as breast and prostate cancers) are less likely to experience fractures across both genders, perhaps in part reflecting the known differences in bone mineral density between the AA and NHW racial groups.[23] Interventions (radiation, bone surgery) were also significantly less likely in AA women (Table 3); the lower likelihood for such interventions may partly be due to the lower occurrence of fractures among the AA patients. However, the overall higher percentage of AA women who were in poorer health (i.e., based on CCI≥2) at diagnosis of advanced BCa compared to NHWs could also have contributed to the lower utilization of radiation and/or bone surgery in the AA group (Table 3). In addition to demographic factors, dietary factors, including calcium and vitamin D intake, behavioral factors such as tobacco abuse and excessive alcohol consumption, and certain other environmental factors and exposures, may directly or indirectly affect bone physiology and potentially alter the risk of skeletal fractures. However, we were not able to include these variables in the analysis as they were not available in the SEER-Medicare dataset.

That ER/PR was unknown in 23% of our patient population seems to represent a relatively large number of women in whom such information was missing given that the current ASCO/CAP (American Society of Clinical Oncology/College of American Pathologists) guidelines strongly recommend testing all BCa tumors for ER/PR. It is unknown whether there were issues with data collection or that ER/PR status was simply not assessed in some women. Notably, there did not appear to be any discrepancies due to race as a similar proportion of AA and NHW women were ER/PR unknown (p = 0.09). Nevertheless, the women identified by ER/PR ‘unknown status’ had a particularly shorter median follow up and higher one-year mortality (p<0.0001), suggesting perhaps a more aggressive disease behavior and/or more advanced metastatic disease at diagnosis among the ER/PR unknown group. The women with unknown ER/PR also had a significantly lower prevalence of SCRT compared to women with known ER/PR status. The shorter follow-up time and the lower percentage of radiation among the ER/PR unknown women likely contributed to the overall lower prevalence of SCRT among this group.

Lastly, we categorized the radiation received in women with an incident diagnosis of advanced BCa as palliative radiation therapy and thus as a SCRT. Included in this category of advanced BCa are women with and without bone metastasis; the use of radiation in this broader patient population may or may not have been directly related to bone metastasis, and hence there is the potential for misclassification of the RAD category as representative of a SRE in the strict context of how radiation-based SREs have been defined in clinical trials. Nevertheless, the present data is informative in providing a benchmark in terms of radiation therapy use in a population of women with metastatic BCa as represented within the SEER dataset. Interestingly, despite the lack of a validated measure of radiation use, a recent study in metastatic prostate cancer patients using SEER-Medicare linked data, in which sites of metastasis within SEER are more well defined (i.e., M1a, M1b, M1c), also found similar patterns with respect to risk factors (age, race, comorbidity index) and radiation use, as well as SCC, PF and BS, in the M1 patient population.[24] We expect that radiation among the breast cancer patients was delivered with full information regarding their cancer stage in most cases. Thus, we believe that it is unlikely that our conclusions regarding the risk factors for a SCRT (including palliative radiation therapy) would change when using a validated measure of radiation therapy.

In summary, the present study has identified patient and disease related factors that are associated with the occurrence of spinal cord compression or pathologic fracture, or necessity for bone surgery or use of radiation (i.e. SCRT) in a general population of older women with incident metastatic BCa. To what extent such factors may also be relevant to women younger than age 66 needs to be determined. This analysis also highlights the need to study further the risk factors associated with the SCRT subtypes. Such information should better clarify the clinical implications and utilization of resources (such as need for radiation, surgery, or hospitalization) related to these SCRT subcomponents, and therefore ultimately facilitate the overall management of women with advanced breast cancers.
